# Complete genome characterization of a rotavirus B (RVB) strain identified in Alpine goat kids with enteritis reveals inter-species transmission with RVB bovine strains

**DOI:** 10.1099/jgv.0.001022

**Published:** 2018-03-08

**Authors:** Fangzhou Chen, Todd P. Knutson, Max Ciarlet, Matthew Sturos, Douglas G. Marthaler

**Affiliations:** ^1^​ Department of Veterinary Population Medicine, College of Veterinary Medicine, University of Minnesota, St. Paul, MN 55108, USA; ^2^​ State Key Laboratory of Agricultural Microbiology, College of Veterinary Medicine, Huazhong Agricultural University, Wuhan 430070, PR China; ^3^​ Vaccines Clinical Research and Development, GlaxoSmithKline Vaccines, Cambridge, MA 02139, USA; ^4^​ Veterinary Diagnostic Laboratory, College of Veterinary Medicine, Manhattan, KS 66506, USA

**Keywords:** goat rotavirus B, interspecies transmission, phylogenetics

## Abstract

Rotavirus B (RVB) has been associated with enteric disease in many animal species. An RVB strain was identified in pooled intestinal samples from Alpine caprine kids (between 2 and 3 days of age) experiencing high (>90 %) morbidity, and the complete caprine RVB genome was characterized. Histology revealed villus atrophy, the samples tested positive for RVB by real-time RT-PCR and metagenomic next-generation sequencing identified only RVB and orf virus. In the VP4 gene segment, the caprine RVB strain had a higher percentage nucleotide identity to the Indian bovine RVB strains than to the Japanese bovine RVB strains, but the VP7, VP6, VP2, NSP1, NSP2 and NSP5 gene segments of the American caprine RVB strain were genetically related to the Japanese bovine RVB strains. The results indicate a lack of RVB sequences to understand reassortment or the evolutionary relationship of RVB strains from cattle and goats.

Rotaviruses (RVs) (genus *Rotavirus*, family *Reoviridae*) are of major global significance as causes of diarrhoea in young animals [1]. The RV genome contains 11 gene segments of dsRNA, including six structural viral protein (VP) genes (VP1–4, VP6 and VP7) and five to six non-structural protein (NSP) genes (NSP1–NSP5/NSP6). The RV icosahedral capsid is composed of three concentric protein layers encoded by VP7 and VP4 (outer layer), VP6 (middle layer) and VP1, VP2 and VP3 (inner layer). The non-structural proteins are associated with virus replication, translation, innate host immune response and morphogenesis [2].

Based on the percentage nucleotide identities of the VP6 gene segment, RVs were clustered into eight species (RVA–RVH) [3]. Currently, two new tentative RV species (RVI and RVJ) have been identified from dogs [4] and bats [5], respectively. RVB strains can cause sporadic or epidemic diarrhoea in humans [6] and a variety of animals, including sheep [7], goats, cows, pigs and rats [8]. In addition, RVB infections have been identified in multiple countries, including Bangladesh [9], Brazil [10], China [6], the Czech Republic [11], India [12], Japan [13], New Zealand [14], Spain [15], the USA [16] and the UK [17], by genetic and/or serological methods.

While several segments of RVB originating from different host species have been investigated, a single porcine and rat and multiple human complete RVB genomes have been reported [18, 19]. In goats, only RVA sequences have been published, and inter-species transmission events have been identified between goats and humans, cows and different camelids [20–22].

Worldwide, there are 861.9 million goats [23], and goats are economically important animals for small farmers, especially in poor, rural regions, because goats provide many products, such as meat, milk, skin for leather, fibre and cheese [24]. In goats, clinical RV infections have been characterized by diarrhoea with pasty to watery faeces, anorexia, dehydration and prostration in young goats [15, 25, 26].

In the spring of 2016, Alpine goats on a caprine farm in Minnesota were experiencing diarrhoea in kids of between 2 and 3 days of age, with very high (>90 %) morbidity but without mortality. The scours within the kids occurred for the entire length of the kidding season. Thus, the owner decided to deliver two 3-day-old goat kids to the University of Minnesota Veterinary Diagnostic Laboratory (UMVDL) for clinical care and to determine the causative agent of the disease. On gross examination, the intestinal and colonic contents were tan to yellow and watery. The intestines were submitted for histopathology, molecular diagnostics and electron microscopy. On histopathology, there was mild segmental degeneration of the villus-tip epithelial cells in the intestine of goat kid 1, and moderate segmental villus epithelial degeneration, rare single-cell necrosis and sloughing, and segmental moderate villus atrophy in goat kid 2 (Fig. 1). Cellular degeneration was characterized by non-staining to pale eosinophilic cytoplasmic vacuoles or cellular attenuation, and necrotic cells had similar cytoplasmic vacuolization with pyknotic or karyorrhectic nuclei. Intestinal homogenates from the individual kids were tested for RVA and RVB by multiplex real-time RT-PCR and were negative for RVA but positive for RVB (kids 1 and 2; *C*_t_ 32.6 and 27.06, respectively), and RV-like virions were detected in the faeces of goat kid 2 by negative stain electron microscopy. Virions were not detected in the faeces of goat kid 1. *Clostridium perfringens* was isolated from the intestine of goat kid 1 in anaerobic culture but was not isolated from the intestine of goat kid 2. There was no significant bacterial growth from the intestine of either goat kid in aerobic culture. *Salmonella* species were not isolated from the colon or pooled tissues of either goat kid in enriched culture.

**Fig. 1.**
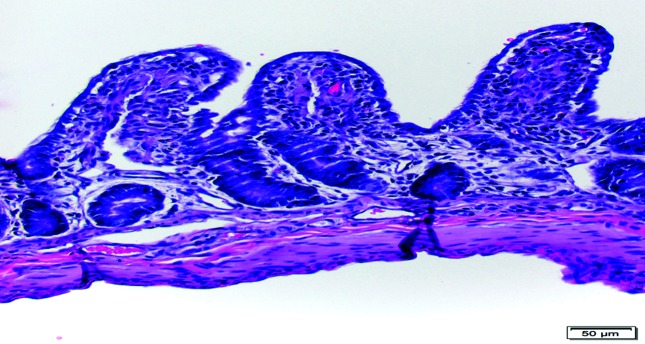
Villus epithelial degeneration and atrophy in goat kid 2 from fixed tissue. Bar, 50 µm.

To investigate the viral infection spectrum in these kids, the RNA from the pooled intestine samples (kids 1 and 2) were combined and submitted to the University of Minnesota Genomics Center (UMGC) for next-generation sequencing (NGS) on the Illumina MiSeq (2×250 bases) as previously described [27]. The raw sequence reads were trimmed for quality and *de novo* assembled using IDBA-UD within A5 software [28, 29], and assemblies were verified by remapping with bowtie2 V2.2.4 [30, 31]. A total of 447 542 reads were generated from the NGS, and only RVB and orf (genus *Parapoxvirus*) viruses were identified [6949 reads (1.55 %) and 15 497 reads (3.46 %), respectively].

The genome of the orf virus could not be assembled due to the limited number of reads. Orf virus is an epitheliotropic virus, which causes pustular dermatitis (contagious ecthyma or ‘sore mouth’) in goats and sheep, and is capable of infecting multiple ruminant and mammalian species [32]. These goat kids lacked skin or oral lesions, and thus, detection of this virus is evidence of environmental exposure or possibly an acute subclinical infection, but it is unrelated to enteritis in these goat kids.

The RVB reads were *de novo* assembled, and the RVB gene segments were deposited into GenBank (KY689687 to KY689697) as RVB/Goat-wt/Minnesota-1/USA/2016. Complete genome characterization is essential to investigate the evolution of RVs as RVs have a natural ability to reassort during coinfections. The sequence alignment for each of the RVB gene segments was constructed using ClustalW in mega v.6 software with the available RVB sequences in GenBank. The nucleotide and amino acid sequence identities were analysed with the Lasergene package MegAlign software v7.1.0 (DNASTAR) (Table 1). The coding sequence length for all the gene segments of the caprine strain was the same as those of the bovine, porcine, human and rat RVB strains, except for the NSP3 gene, in which the caprine strain was 153 nt shorter than the human strains and 207 nt shorter than the rat strain. The caprine RVB strain shared the highest percentage nucleotide and amino acid identities with bovine RVB strains (71.0–98.1 % and 71.3–98.1 %, respectively) while the nucleotide and amino acid sequence identities of caprine RVB genes ranged from 43.3 to 77.6 % and 34.4 to 72.6 %, respectively, compared with those of porcine, human and rat RVB gene segments (Table 1).

**Table 1.** Comparison of segment size and nucleotide and amino acid sequences identities (percentages) of the caprine RVB strain with those of the bovine, porcine, human and rat RVB strains

Gene segment	Bovine		Porcine		Human		Rat	
	nt	aa	nt	aa	nt	aa	nt	aa
VP7 (744, 247)	77.3–98.1 (744)	72.0–98.1 (247)	58.3–69.9 (747, 750)	38.3–61.9 (247, 248)	61.6–63.2 (750)	46.4–50.0 (249)	59.6 (741)	53.1 (246)
VP4 (2277, 758)	89.1–89.6 (2277)	93.0–93.4 (758)	68.4 (2250)	60.4 (749)	58.8–59.3 (2253)	55.9–57.5 (750)	65.6 (2256)	71.7 (751)
VP6 (1176, 391)	82.0–93.6 (1176)	79.0–93.2 (391)	65.1–75.5 (1176)	52.6–70.1 (391)	66.0–67.0 (1176)	43.7–45.7 (391)	66.0 (1176)	72.2 (391)
VP1 (3477, 1158)	76.7–76.8 (3477)	71.3–71.4 (1158)	64.9 (3483)	52.4 (1160)	64.0–64.4 (3483)	50.5–51.3 (1160)	63.5 (3480)	67.6 (1159)
VP2 (2814, 937)	77.5–77.6 (2814)	72.6–72.8 (937)	68.5 (2805)	71.1 (934)	64.7–66.3 (2802, 2805)	52.2–55.2 (933, 934)	65.9 (2805)	68.9 (934)
VP3 (2292, 763)	94.5–94.9 (2292)	94.3–94.9 (763)	64.8 (2292)	61.5 (763)	63.9–64.4 (2292)	50.1–51.2 (763)	64.0 (2292)	61.9 (763)
NSP1-1(306, 101)	76.7–97.5 (306)	72.3–97.0 (101)	61.2–66.5 (306)	51.5–53.5 (101)	46.0–48.4 (324)	39.6–41.6 (107)	49.1 (348)	41.2 (115)
NSP1-2(963, 320)	71.0–96.7 (963)	72.3–93.5 (320)	60.2–61.9 (963)	53.0–57.3 (320)	51.5–53.3 (966)	44.3–45.2 (321)	53.5 (963)	45.0 (320)
NSP2 (903, 300)	80.4–92.6 (903)	76.7–92.0 (300)	68.3–77.6 (903, 906)	52.7–71.7 (300, 301)	68.0–69.0 (906)	58.3–60.1 (301)	68.4 (906)	73.3 (301)
NSP3 (891, 296)	94.4–95.2 (891)	95.3–97.0 (296)	48.3 (849)	39.7 (282)	43.4–45.3 (1044)	41.9–43.9 (347)	45.6 (1098)	36.1 (365)
NSP4 (627, 208)	93.0–94.2 (627)	95.3–97.0 (208)	49.8 (657)	35.8 (218)	48.3–49.7 (660)	34.4–34.9 (219)	49.8 (657)	31.6 (218)
NSP5 (501, 166)	78.6–95.6 (501)	85.0–98.8 (166)	48.7–75.2 (504, 522, 525, 528)	42.9–72.6 (167, 173, 174, 175)	49.8–50.8 (513)	46.9–48.6 (170)	56.4 (525)	45.5 (174)

The length of coding sequence nucleotides and amino acids are listed in parentheses.

Partial bovine RVB genomes have been reported from Japan and India (Table 2). The caprine VP7, VP6, VP2, NSP1, NSP2 and NSP5 gene segments had a higher percentage nucleotide identity to the cogent Japanese bovine RVB gene segments than to the Indian bovine RVB gene segments, except in the VP4 gene segment, which illustrates a Japanese bovine RVB genome constellation, with a Japanese VP4 gene segment.

**Table 2.** Nucleotide percentage identities of the caprine RVB strain to the bovine strains from India and Japan

	VP7	VP4	VP6	VP1	VP2	VP3	NSP1-1	NSP1-2	NSP2	NSP3	NSP4	NSP5
Indian	77.2–72.3 %	89.5–89.9 %	82.0–82.5 %	76.70 %	77.1–77.3 %	na	75.5 %	70.9–71.4 %	80.3–81.3 %	na	na	77.4–78.2 %
Japanese	92.3–98.1 %	81.4–82.2 %	93.60 %	na	90.4–90.6 %	91.3–95.1 %	94.8–97.4 %	93.0–96.7 %	92.6 %	94.4–95.3 %	92.7–93.9 %	95.0–95.4 %

Higher nucleotide identities were shaded; na, Bovine RVB sequences were not available from that region.

Rotavirus Classification Working Group (RCWG) recommendations were developed and widely applied in RVA classification, but classification for RVB has not been developed for the 11 gene segments [33]. Recently, the nucleotide cut-off values of 80, 81, 70, 78 and 70 % were used to genotype the VP7, VP6, VP3, NSP3 and NSP4 gene segments of RVB strains, respectively [13, 16, 34]. The NSP1 and NSP5 gene of RVB were also divided into seven and six clusters [13], respectively. The classification methodology is hindered due to the limited number RVB strain sequences.

Phylogenetic trees were reconstructed using the maximum-likelihood algorithm, with the GTR nucleotide substitution model (bootstrap analysis with 1000 replicates) with mega v.6 software. In the phylogenetic trees for each of the 11 gene segments (Fig. 2a–k), the caprine RVB strain shared a common ancestor with the bovine RVB strains from Japan, except in the VP4 gene segment, which shared a common ancestor with the bovine RVB strains from India. These phylogenetic analyses were consistent with the above nucleotide identity analyses, suggesting an inter-species transmission between caprine and bovine RVB strains. Further studies are needed to understand the genetic and interspecies relationships between caprine and bovine RVB strains.

**Fig. 2.**
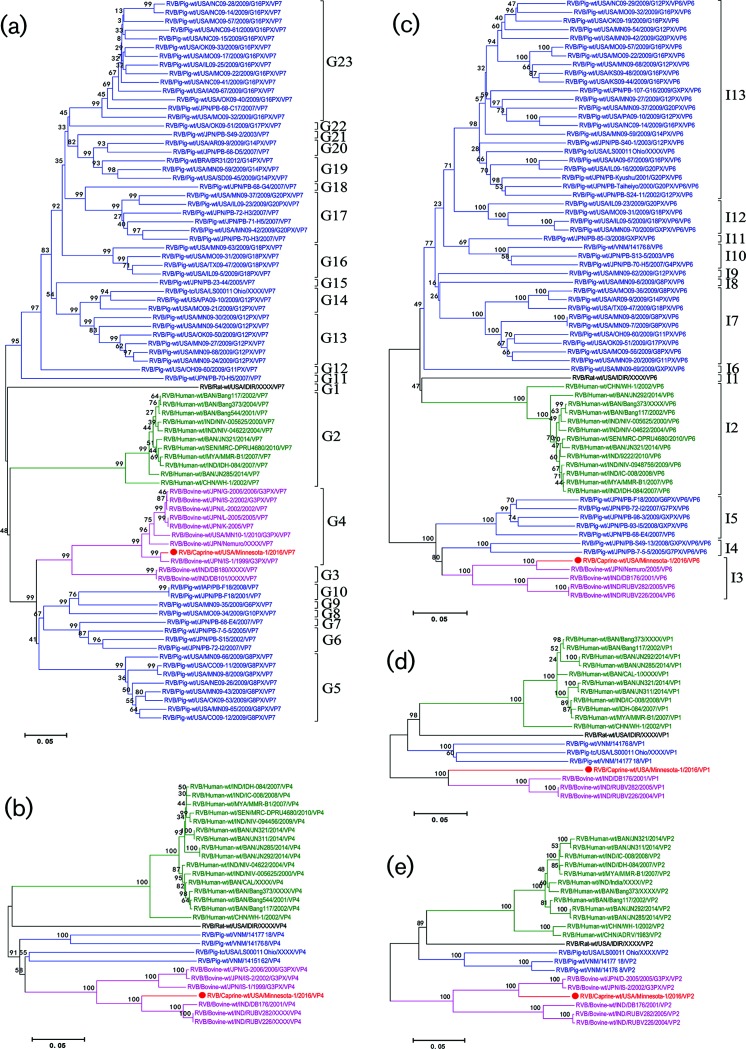

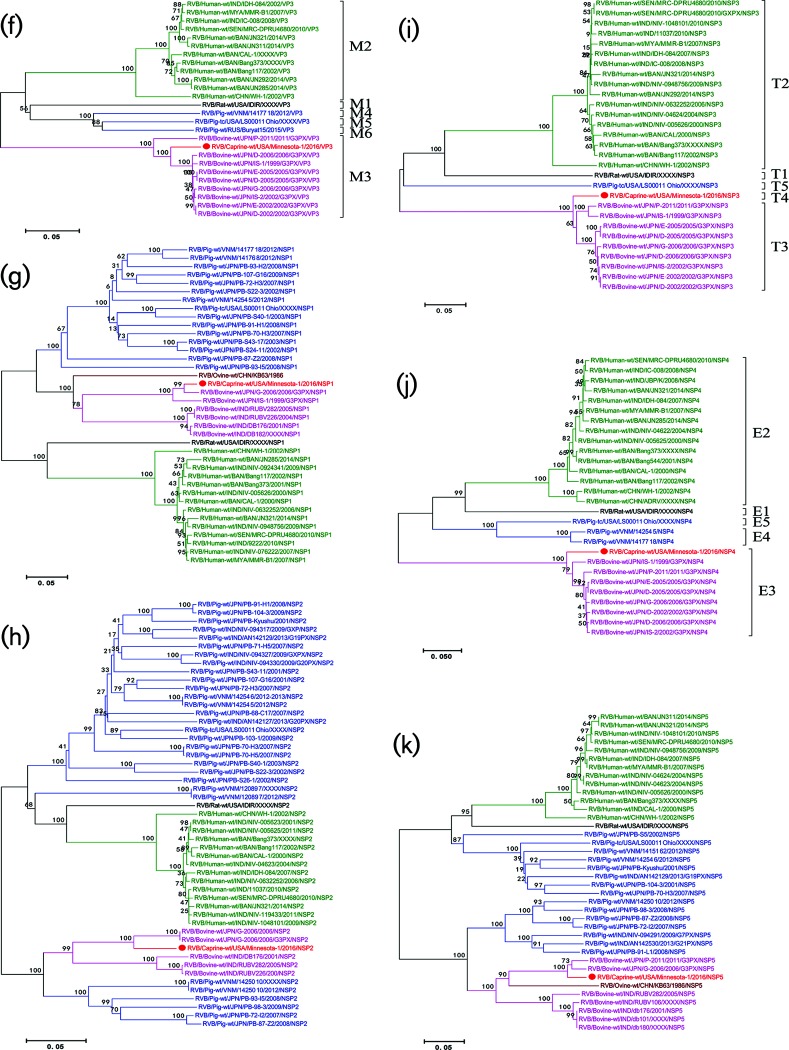
Phylogenetic trees of different RVB gene segments, VP7 (a), VP4 (b), VP6 (c), VP1 (d), VP2 (e), VP3 (f), NSP1 (g), NSP2 (h), NSP3 (i), NSP4 (j) and NSP5 (k), of the study strain with cognate gene segments of the other RVB strains. Red circles indicate the different segments of the USA RVB strain: RVB/Goat-wt/USA/Minnesota-1/2016. The ovine, rat, bovine, pig and human rotavirus strains are highlighted in colours maroon, black, fuchsia, blue and green, respectively. Bars, 0.05 nucleotide substitutions per site.

In conclusion, this study identified RVB as the causative agent of enteritis in goat kids through the detection of rotavirus-like virions in the faeces of goat kid 2 and confirmation with positive RVB PCR of the intestinal contents. The lack of detection of virions in the faeces of goat kid 1 may indicate that the infection was in the pre-latent phase or that the amount of virus being shed in the faeces was below the threshold of detection by negative stain electron microscopy. The significance of the *Clostridium perfringens* isolated from the intestine of goat kid 1 is uncertain as this bacterium may be isolated from non-diarrhoeic goats. In addition, the complete caprine RVB genome was characterized using metagenomic NGS. The nucleotide sequences and phylogenetic analyses suggested the possible inter-species transmission between caprine and bovine RVB strains. Further studies should screen RVB in caprine and bovine herds to understand this evolutionary relationship within ruminants.
